# Halotolerant Ability and α-Amylase Activity of Some Saltwater Fungal Isolates 

**Published:** 2013

**Authors:** Farhad Niknejad, Mahsa Moshfegh, Mohammad Javad Najafzadeh, Jos Houbraken, Shahla Rezaei, Gholamreza Zarrini, Mohammad Ali Faramarzi, Nastaran Nafissi-Varcheh

**Affiliations:** a *Laboratory Sciences Research Center, Golestan University of Medical Sciences, Gorgan, Iran.*; b *Department of Pharmaceutical Biotechnology, Faculty of Pharmacy and Biotechnology Research Center, Tehran University of Medical Sciences, Tehran, Iran.*; c *Department of Parasitology and Mycology, and Cancer Molecular Pathology Research Center, Ghaem Hospital, School of Medicine, Mashhad University of Medical Sciences, Mashhad, Iran.*; d *Department of Applied and Industrial Mycology, CBS-KNAW Fungal Biodiversity Centre, Utrecht, The Netherlands.*; e *Department of Animal Sciences, Faculty of Natural Sciences*, *University of Tabriz, Tabriz, Iran*; f ^f^*Department of Pharmaceutical Biotechnology, School of Pharmacy and Protein Technology Research Center, Shahid Beheshti University of Medical Sciences, Tehran, Iran.*

**Keywords:** Isolation, Amylase, Halotolerant, Fungi, Hypersaline environments

## Abstract

Four halotolerant fungal isolates originating from the saltwater Lake Urmia in Iran were selected during a screening program for salt resistance and *α*-amylase activity. The isolates were identified based on sequencing the ITS region and a part of the *β*-tubulin gene, as *Penicillium chrysogenum *(isolate U1; CBS 132820), *Fusarium incarnatum *(isolate U2; CBS 132821), and *Penicillium polonicum *(isolate U3; CBS 132822, and isolate U4; CBS 132823)*. *The growth of these isolates was determined by measuring the colony diameter and mycelia dry weight in Sabouraud dextrose agar and yeast nitrogen base medium supplemented with NaCl, KCl, and LiCl. Isolate U4 showed a growth up in 15% NaCl and U1 was the only isolate that could grow in 20% KCl. None of the strains grew in a media containing LiCl. The salt supplemented medium did not increase the size of colony diameter in all isolates (p > 0.05). The ability of the selected isolates for amylase production was quantitatively tested and showed that *P. polonicum *isolate U4 was the most potent producer of amylase with a yield of 260.9 U/L after 60 h, whereas *P. polonicum *isolate U3 was the lowest one with a production level of 97.9 U/L after 48 h. *P. polonicum *isolate U4 could be a suitable candidate for production of amylase on an industrial scale after optimization.

## Introduction

Extremophilic microorganisms can thrive in extreme environments such as unusual levels of salt, pH, pressure, temperature, *etc.*, and those which are adapted to live in hypersaline habitats are considered halophiles (1). The halophilic microorganism able to survive in the presence as well as in the absence of salt is categorized as halotolerant (2). Halophiles represent valuable sources of various biomolecules which can offer potential applications for biocatalysis and biotransformation (3). Most of the studies involving halophilic and halotolerant microorganisms have focused on halophilic bacteria and archaea, whereas there have been a few reports on halophilic and halotolerant fungi communities (4). Black yeasts were the first documented fungi that form an active population in hypersaline seawater (5). Studies on the fungi in hypersaline environments have showed the abundant and consistent occurrence of some specialized fungal species (4). One of the major reasons for the special interest in the extremophiles is to provide the opportunity for harsh industrial processes performed by their robust enzymes (6, 7).

Urmia Lake is the largest lake in the Middle East, located in the northwestern Iran, and the third largest salt water lake on the earth, with a surface area of approximately 5,200 Km², 140 km length, 55 Km width, and 16 m depth. The concentration of NaCl was 34 g/L in 1915 and it has risen to more than 300 g/L due to drought, evaporation and increased agricultural of water use (8). The biota of Lake Urmia is restricted (9) to a few hyperhalophilous phytoplankton green alga (*Dunaliella*, *Nitzschia*, and *Navicula*), the crustacean macrozooplankton such as *Artemia urmiana *and halophilic or halotolerant bacteria and fungi.

α-Amylases (1,4-*α*-D-glucan glucanohydrolase, EC 3.2.1.1) belong to the class of hydrolytic enzymes (10-15), which acts an essential role in hydrolysis of starch in textiles, baking, food, brewing, laundry, as well as in the pharmaceutical industries (16, 17). Until now, numerous starch-hydrolyzing enzymes have been reported in bacteria and fungi; however, few researches are available on starch and amylose mobilization by halotolerant fungal *α*-amylases.

The present study describes the characteristics of some halotolerant fungi, which were isolated from the saltwater Lake Urmia, Iran. Furthermore, their potential for amylase production was investigated.

## Experimental

*Chemicals*

Casamino Acids (CSM) obtained from Obiogene (Carlsbad, CA, USA). 3,5-Dinitrosalicylic acid (DNS) and sodium potassium tartrate were purchased from Sigma-Aldrich (St. Louis, Mo, USA). Starch, yeast nitrogen base medium (YNB), Sabouraud dextrose agar (SDA), and other chemicals were obtained from Merck (Darmstadt, Germany).

*Screening for amylase-producing fungi*

The isolates of filamentous fungi were collected from different locations, at the surface and various depths of solar salt, saline mud from Urmia Lake (37°42′N and 45°19′E), Urmia, Iran. Halotolerant ability of fungi was followed in SDA supplemented with different concentrations of NaCl. The selected fungi were also examined for amylase activity in starch agar medium, which contains the following (g/L): NaCl, 50; K_2_HPO_4_, 1; MgSO_4_, 1; yeast, 5; starch, 10; agar, 15. After the incubation at 27 ºC for 14 days, the amylolytic activity was assessed by flooding the plates with 0.3% I_2_-0.6% KI solution (18). The ratio of clear zone around the growth to colony size was used as an indicator to select the amylase-producing isolates. Four halotolerant fungi with biggest clear zone were selected for more investigations.

*Phylogenetic and phenotypic analysis of the halotolerant fungi*

Total genomic DNA was extracted and stored as described by Houbraken *et al. *(19). The ITS region and a part of the *β*-tubulin gene were amplified and sequenced according the method described previously (20). A BLAST search with the obtained sequences was performed in internal databases of the CBS-KNAW Fungal Biodiversity Centre. Phenotypic studies were also performed according the methods described by Starkey *et al. *(21) and Tiwari *et al. *(22).

*Growth of fungal isolates on different concentrations of NaCl, KCl, and LiCl*

Spore-suspension containing 1×10^6^ conidia per mL were prepared and a volume of 20 μL of each selected strain was inoculated in a spot on 10 mm diameter Petri-dishes containing SDA supplemented with 0, 5, 10, 15 and 20% (w/v) NaCl, KCl, and LiCl. All plates were incubated at 27 ± 1°C and growth was followed by measuring the colony diameter after 2, 5, 7, and 10 days. 

*Effect of different salts on fungi mycelial dry weight*

For determination of the growth rate, the isolates were cultured in two parallels in a defined liquid medium as follows: YNB, 1.6 g/L; CSM, 0.8 g/L; (NH_4_)_2_SO_4_, 5 g/L; and glucose, 20 g/L. This medium was supplemented with different concentrations of NaCl and KCl. Incubation was performed at 27 ± 1°C on a rotary shaker at 180 rpm. Prior to inoculation, the cultures were grown to mid-exponential phase in 50 mL liquid YNB media with the same concentrations of NaCl and KCl. A total of 1 mL of the inoculum was added to 100 mL of YNB (with the same concentration) in 500 mL Erlenmeyer flasks for sample cultures (inoculum was 1% of the final culture volume). Mycelial dry weight was assessed every three days. The dry biomass weight was determined after filtration through dry pre-weighed filter papers, washed three times with distilled water for medium removing, and subsequently dried at 60-70°C to a constant weight.

*Production of amylase in submerged culture*

Amylase production was performed by inoculating 1 mL spore suspension (10^4^ conidia per mL medium) in 250 mL Erlenmeyer flasks containing 50 mL of liquid medium (50 g/L NaCl, 1 g/L K_2_HPO_4_, 1 g/L MgSO_4_, 5 g/L yeast and 10 g/L starch) and subsequent incubation for 7 days at 27 ± 1ºC. The supernatants were assayed for amylase activity every 24 h.

*Amylolytic activity assay*

Cell-free supernatants collected by centrifuging for 20 min at 5,500 ×g at 4ºC were analyzed for amylase activity according to the method described by Miller (23). The reaction mixture containing 450 μL enzyme and 450 μL of 1% (w/v) soluble starch in 50 mM phosphate buffer, 5% (w/v) NaCl, with pH of 7.5 was incubated at 60ºC and 100 rpm for 30 min. The amount of reducing sugar was quantified by DNS with glucose as the standard. One unit of activity was defined as the amount of enzyme that released 1 μmol of reducing sugars per minute under the assay conditions.

*Effects of salt concentration, temperature, and pH on the amylase activity*

The dependence of amylolytic activity to salt concentration was determined in a broad range of NaCl concentrations (0-20% w/v). The study of the effect of temperature was investigated at the temperature range of 50-90ºC. The effect of pH values were determined in 50 mM citrate buffer (pH of 3-5), 50 mM phosphate buffer (pH of 6-7), and 50 mM Tris-HCl (pH of 8-9).

*Statistical analyses*

All the experiments were performed in triplicates and data are mean of three independent measurements with significance of p ≤ 0.05.

## Results

The phylogenetic analyses of the isolates were performed by comparison of ITS and *β*-tubulin gene sequences with those available in the internal database of the CBS-KNAW Fungal Biodiversity Centre. The isolates were identified as *Penicillium chrysogenum *(U1), *Fusarium incarnatum *(U2), and two strains of *Penicillium polonicum *(U3 and U4). The strains were submitted in the culture collection of the CBS-KNAW Fungal Biodiversity Centre, Utrecht, Netherlands under accession numbers CBS 132820 (isolate U1; *P. chrysogenum*), CBS 132821 (isolate U2; *F. incarnatum*), CBS 132822 (isolate U3; *P. polonicum*), and CBS 132823 (isolate U4; *P. polonicum*). The phenotypic characterizations of the fungi were also in accordance with the genetic analyses results.

**Figure 1:**
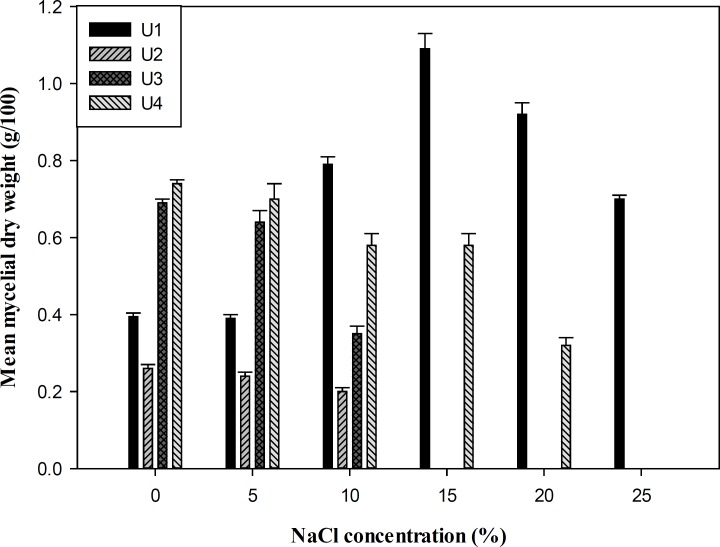
*Mean mycelial dry weigh of fungal isolates (g/100 mL YNB medium) after 12 days* ***.*** *U1: *P. chrysogenum *CBS 132820*, *U2: *F. incarnatum *CBS 132821, U3 and U4: *P. polonicum *CBS 132822 and CBS 132823*.

**Figure 2:**
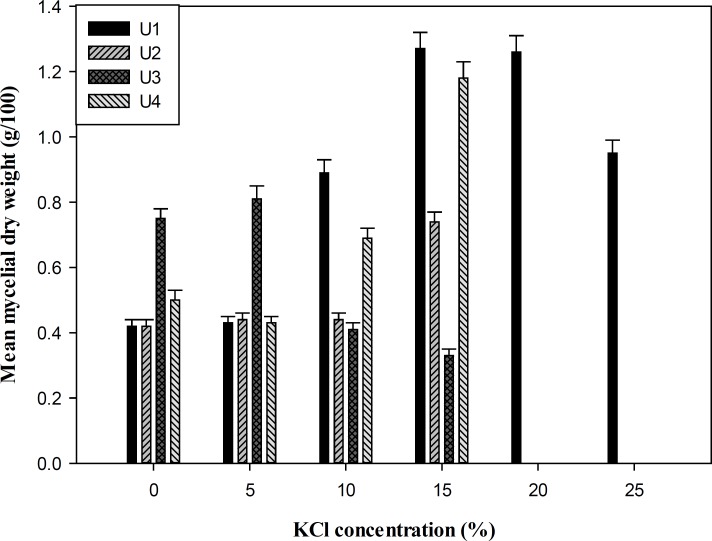
*Mean mycelial dry weigh of fungal isolates (g/100 mL YNB medium) after 12 days* ***.*** *U1: *P. chrysogenum *CBS 132820*, *U2: *F. incarnatum *CBS No. 132821, U3 and U4: *P. polonicum *CBS 132822 and CBS 132823*.

Measurement of mycelial dry weight showed that all the isolates were able to grow at salinity ranging from 0-10% (w/v) NaCl and 0-15% (w/v) KCl in YNB media, whereas *P. chrysogenum *(U1) was the most tolerant isolate that grew up to 25% salinity with the optimal growth and biomass production at 15% (w/v) salt concentration (Figures 1 and 2). To study the effect of salts on the growth of the fungi, the isolates were grown at different concentrations of NaCl, KCl, and LiCl (0-20%) in SDA media. Overall, the salt supplemented medium could not increase the size of colony diameter in all isolates (p > 0.05). The growth rate of the isolates started decreasing from 10% (w/v) NaCl and KCl, and no growth was observed at 20% (w/v) salt concentration except isolate U1. LiCl showed maximum inhibition on growth and none of the strains grew in media containing LiCl (Table 1). 

**Table 1:** Mean diameter of fungal colony growth (millimeter) of selected halotolerant fungi at different concentrations of NaCl and KCl after 10 days.

**Salt concentration % **	**NaCl **				**KCl **			
	**Isolate U1 **	**Isolate U2 **	**Isolate U3 **	**Isolate U4 **	**Isolate U1 **	**Isolate U2 **	**Isolate U3 **	**Isolate U4 **
0	32.75	40	23.75	23	32.75	40	23.75	23
5	33.5	35.75	10	19	37.5	34.25	14.25	37.75
10	25	7.75	12.5	17.75	31	17.5	14.5	28.5
15	14	0	0	13	21.25	9	15	0
20	0	0	0	0	8.5	0	0	0
								

U1: *P. chrysogenum *CBS 132820*, *U2: *F. incarnatum *CBS 132821, U3 and U4: *P. polonicum *CBS 132822 and CBS 132823*. *Values are an average of two sets of experiments (mm).

**Figure 3:**
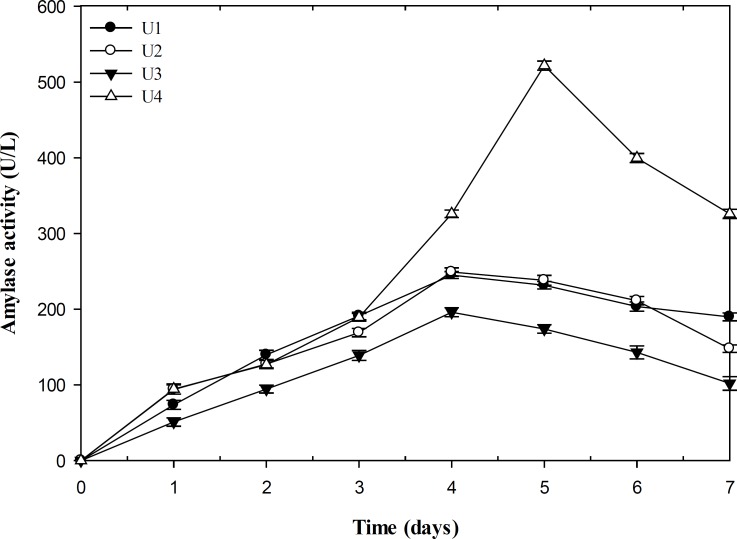
*Comparison of amylase activity in fungal isolates (U/L). U1: *P. chrysogenum *CBS 132820*, *U2: *F. incarnatum *CBS 132821, U3 and U4: *P. polonicum *CBS 132822 and CBS 132823*

The ability of producing amylase was quantitatively tested among selected isolates (Figure 3). The result of flask fermentation indicated that *P. polonicum *(U4) was the most potent producer of amylase with maximal yield of 521.8 U/L after 5 days incubation, whereas the other isolate of *P. polonicum *(U3) was the lowest producer with maximum enzyme production of 195.8 U/L after a time course of 4 days. The influences of pH, temperature, and NaCl concentration on the amylase activity of the isolates were also determined. The strain U1 and U4 showed the optimal amylase activity at pH of 7.5. However, the maximal activities of strain U2 and U3 were detected at 8. The effect of temperature on the enzyme activity indicated a narrow range of optimal temperature at 60ºC for the isolates U1, U2, and U4 and 55ºC for U3. The measurement of the crude enzyme activity in the NaCl concentrations range between 0 and 25% (w/v) revealed the enzyme activity of strain U1 increased up to 10% salt concentration and then decreased. However, in the case of other isolates, maximum activity was determined at 5% NaCl concentration. 

## Discussion

In a preliminary screening of fungal isolates of Urmia Lake, four strains were selected for further investigations according to the size of clear zone around the colonies on starch-agar plates. On the basis of the phenotypic characteristics and comparison of ITS and *β*-tubulin gene sequences, the isolates were identified as *P. chrysogenum *(U1), *F. incarnatum *(U2) and two strains of *P. polonicum *(U3 and U4). Halotolerance of the strains was studied by measurement of mycelia dry weight and colony growth diameter in a wide range of salts. All the isolates could grow in the liquid medium containing (0−10%) NaCl or (0−15%) KCl. However, *P. chrysogenum *(U1) showed the most biomass production at high salinity. This suggests better adaptation of this fungus to high NaCl concentration in the growth medium. The optimal growth of the fungi on SDA medium supplemented with salts was in a range of 0-5% NaCl or KCl. Although there was no clear relationship between the growth patterns of the isolates on SDA medium and liquid medium containing different concentrations of NaCl or KCl, halotolerant properties of the strains were obviously observed. None of the isolates could grow on the media supplemented with LiCl that indicating maximal inhibition at all concentrations of the mentioned salt. Li^+^ had a growth-inhibitory effect on *Debaryomyces nepalensis *NCYC 3413, the halotolerant yeast, by inhibition of some steps in the metabolism of either phosphoinositide or sulfate (24). The toxicity of lithium on *Saccharomyces cerevisiae *has been attributed to the inhibition of phosphoglucomutase activity by Li^+^, which shift the cellular calcium homeostasis and signaling (25). Since the isolates did not show absolute requirement for salt to grow but could adapt to grow at high salt concentrations, they are categorized as halotolerant. Recent studies have shown a considerable diversity and abundance of fungi in natural hypersaline environments worldwide, contrary to the general belief of a lack of halophilic and halotolerant mycobiota in such habitats (4). Although several species of *Penicillum *and *Fusarium *have been previously isolated from hypersaline environment (26, 27), no halotolerant ability of *Fusarium incarnatum *has been reported so far. Halophilic fungi apply several adaptation mechanisms to survive in hypersaline environments (4). Most yeast and fungi produce and accumulate different polyols such as ribitol, erythritol, mannitol, xylitol, sorbitol, and free amino acids to restrict the accumulation of Na^+^ below the toxic levels inside the cells (4). Another kind of adaptation to the stress condition is obtained by alteration in membrane lipid properties. The membrane composition shows a considerable increase in unsaturated phospholipid fatty acids-to-sterol ratio to achieve more fluidity over a wide range of salt concentrations (4, 28). Amylase production by strain U4 was higher as compared to the other isolates. There are a few reports available on the amylase production by halotolerant and halophilic fungi (29, 30). Amylases represented optimum activity at alkaline pH values. Amylases produced by most of fungi have an optimal pH at a range of 4-5. However, two alkaline amylases from halotolerant *Penicillium *sp. have been reported by Gounda and Elbahloul (29) with the optimal pH values of 9 and 11. Maximal activity of amylases was within the range of 55-60ºC. Nwagu and Okolo (31) have studied amylase activity of a thermophilic fungus *Aspergillus fumigatus *with the optimal temperature at 60ºC. The optimal NaCl concentration was accordance to the halotolerant characteristics of the isolates. 

## Conclusion

Although *α*-amylase has been derived from several fungi, yeasts and microbial sources, industrial production of this enzyme has been restricted to only a few strains of bacteria and fungi. Fungal amylases are preferred over other microbial sources due to their more acceptable properties in various food industries. It appears that selected halotolerant and halophilic species synthesize enzymes such as amylase under conditions that represent stress for non-adapted species.
